# Difficulties with Emotion Regulation during COVID-19 and Associations with Boredom in College Students

**DOI:** 10.3390/bs12080296

**Published:** 2022-08-20

**Authors:** Elizabeth H. Weybright, Erica L. Doering, Sammy Perone

**Affiliations:** Department of Human Development, Washington State University, Pullman, WA 99164, USA

**Keywords:** boredom, self-regulation, COVID-19 pandemic

## Abstract

COVID-19 related restrictions resulted in a significant lifestyle change for many young adults in the United States. Although boredom and emotional self-regulation are clearly connected in empirical research, the question remains of what this association looks like in unique circumstances, such as early in COVID-19 pandemic at the height of restrictions. The purpose of the current study is to identify the association between boredom proneness and emotion regulation in college students during the COVID-19 pandemic. College students who completed a Boredom Coping Survey between October and December 2019 (*n* = 481) were recruited for a follow-up COVID-19 Boredom Survey in April 2020. Data from this sub-sample (*n* = 58) were used in a hierarchical regression predicting the role of boredom proneness on COVID-19 pandemic emotion regulation difficulties while controlling for age, sex, and COVID-19 related lifestyle changes. Findings indicated higher levels of emotion regulation difficulties were associated with higher levels of boredom proneness above and beyond demographic variables and COVID-19 lifestyle changes. Results are in line with prior theory and research on the importance of the environment or situational factors to the experience of boredom.

## 1. Introduction

COVID-19 was officially declared a pandemic by the World Health Organization on March 11, 2020 [1] and what followed were a variety of national and local restrictions to prevent spread of the disease. Shortly thereafter, within the United States, individual states issued restrictions, such as stay at home orders, which shut down in-person services including public schools, restaurants, and recreation facilities and spaces. Preliminary research on the impact of COVID-19 related restrictions on people indicates they were associated with negative social and psychological outcomes such as loneliness, stress, and depression [2,3,4].

Early in the pandemic, at a time when most stay at home orders were in place, individuals responded to restrictions with reports of feeling, or expecting to feel, bored. This is evident in public data sources, like Google Trends, where the peak popularity of the term “bored” for the year 2020 was the week of March 29 to April 4 [5]. This range of days is within the most limiting state or local restrictions. This is also evident in emerging research on the pandemic indicating individuals most compliant with COVID-19 related restrictions as well as countries with greater restriction of movement were more likely to report boredom [6].

Boredom is often defined as the desire, yet inability to, engage in meaningful and satisfying activity [7]. Conceptualized as a negative emotion, it can come from feelings of dissatisfaction with one’s environment (e.g., external) or difficulty paying attention (e.g., internal) [8]. van Tilburg and Igou [9] suggest the key distinguishing feature separating boredom from other negative emotions, like frustration, is the lack of meaning.

Westgate and Wilson [10] proposed the Meaning and Attention Components (MAC) model of boredom. This model posits boredom emerges in tasks perceived to have little meaning or tasks that are over or under stimulating, such as tasks that are too easy or too stimulating and difficult. The widespread experience of boredom at the onset of the COVID-19 pandemic is consistent with the MAC model. For example, people experienced a drastic change in environment with fewer social interactions and restricted access to leisure activities, reducing stimulation and the autonomy to create stimulation as in pre-pandemic life, leading to more boredom. 

From a functional perspective, boredom serves to tell us that our current environment is not what we want it to be and motivates us to make behavioral or cognitive changes [11]. However, individuals differ in their ability to use emotional cues, such as boredom, to adapt behavior to match the demands of the environment. Boredom as a cue to redirect attention is the centerpiece of the Boredom Feedback Model (BFM) proposed by Tam et al. [12]. In the model, boredom serves as cue to redirect attention to external and internal sources that can, in turn, lead to new opportunities for behavior, thereby mitigating boredom. 

Almost everyone experiences situational (i.e., state) boredom at some point in their lives [13]. However, approximately 10–20% of people experience boredom more frequently than others [14,15] which may reflect an inability to effectively use boredom as a cue to redirect attention to a more satisfying activity. This chronic experience of boredom is referred to as boredom proneness, one type of trait boredom often measured via self-report [16]. People high in boredom proneness are more likely to experience dysregulation, such as anxiety and depression, and are more sensitive to punishment [8]. Perone et al. [17] found people who vary in boredom proneness engage different styles of self-regulation when they experience boredom. They used electroencephalography (EEG) to obtain neural signatures of self-regulation during a repetitive task designed to induce boredom. They found people low in boredom proneness exhibited a pattern of brain activity associated with creating stimulation to cope whereas people high in boredom proneness exhibited a pattern of brain activity associated with a desire to withdraw and stress. Put differently, people low in boredom proneness more adaptively matched their response to the task than people high in boredom proneness. Trait and state boredom also interact. For example, Weybright et al. [18] found adolescents who were higher in trait boredom were more likely to use substances while also experiencing higher state boredom.

Based on Self-Determination Theory, COVID-19 related restrictions and consequences of non-compliance can be considered autonomy controlling environments, or settings which thwart an individual’s belief their actions are self-determined [19]. Reductions in autonomy or perceived environmental constraints, like those during COVID-19, especially early, are associated with experiences of boredom [20]. With the sudden shift to a controlling environment with limited access to leisure pursuits outside the home as well as social opportunities, individuals experienced significant lifestyle changes, likely requiring use of self-regulatory processes in a novel environment. Self-regulation is the process of aligning behaviors with one’s goals (i.e., goal-directed behavior) [21] and plays a central role in regulation of emotions, including boredom. Individuals who are better able to regulate their emotions are likely those low in boredom proneness due to their ability to satisfactorily resolve feelings of boredom. However, even individuals who regulate their emotions well were likely challenged by the novel COVID-19 environment. 

The COVID-19 pandemic and related restrictions resulted in a significant lifestyle change for many young adults in the United States. Although boredom and emotion regulation are clearly connected in empirical research [8], the question remains of what this association looks like in unique circumstances, such as early in the COVID-19 pandemic at the height of restrictions. Prior studies have shown people low in boredom proneness are able to effectively adapt their response when completing tasks designed to induce boredom, an indicator of effective emotion regulation [17]. We hypothesize individuals who are less able to effectively cope with or apply strategies to resolve negative emotions, such as boredom, would experience boredom more frequently. To test this hypothesis, we measured emotion regulation abilities using the Difficulties in Emotion Regulation–Short Form (DERS-SF) [22]. This scale captures several aspects of emotion regulation abilities that are important for mitigating negative emotions, such as boredom, including perceived access to effective emotion regulation strategies and ability to engage in goal-directed behavior. We expected difficulties in emotion regulation at the onset of the pandemic would be associated with higher levels of boredom proneness. Better understanding emotion regulation and the association with boredom will provide insight into what strategies may be useful in promoting effective boredom coping. Given preliminary research indicating compliance with COVID-19 related restrictions is associated with boredom, we controlled for these as COVID-19 lifestyle changes, in addition to demographics of age and sex. 

## 2. Materials and Methods

The sample for the current study came from undergraduate students (*n* = 481) who completed a College Student Boredom Coping Survey between October and December 2019. The survey was anonymous, but participant emails were collected for distribution of incentives. These emails were used to recruit for a follow-up COVID-19 Boredom Survey. Therefore, inclusion criteria for this follow-up survey was participation in the College Student Boredom Coping Survey. Between 7 April and 19 April 2020, 86 undergraduate students initiated the survey for a $5 Amazon e-gift card. Students who did not complete the majority of the survey (i.e., >90%; *n* = 20) or who answered both attention checks incorrectly were removed (*n* = 2). The sample was limited to students on-campus, to focus on students who shared the most similar environments pre-pandemic and early in the pandemic when this study was conducted, and therefore excluded online-only students (*n* = 6). Students on campus had the shared experience of attending class in person and then switching to a remote learning environment. Therefore, the final sample consisted of 58 students. Within this sample, the mean age was 19.78 (SD = 1.55), most participants (87.9%) identified as female for sex, and 53.4% identified as White, 13.8% Multi-racial, 13.8% Hispanic or Latino, 12.1% Asian or Asian American, 3.4% Native Hawaiian or Other Pacific Islander, 1.7% American Indian or Alaska Native, and 1.7% Black or African American for racial/ethnic identification. This study was reviewed and approved by the author-affiliated Institutional Review Board. Informed consent was obtained from all subjects involved in the study.

### Measures

See Table 1 for scales or items used to reflect key constructs, response options, and related descriptive statistics and Supplementary Table S1 for each scale item and response option. The Short Boredom Proneness Scale (BPS-SR) is an 8-item scale (e.g., “I find it hard to entertain myself”) was used to measure boredom proneness [23]. To reflect boredom experiences in the COVID-19 pandemic, the prompt of “Thinking of yourself over the past two weeks, select the phrase that best describes the degree to which you agree or disagree with each statement” was provided to participants prior to answering the scale items. Higher scores reflect higher boredom proneness. Scale reliability was good, consistent with prior reliability [23].

Three items were used to capture COVID-19 related lifestyle changes including practicing physical distancing, engagement in safe outdoor shelter-in-place activities, and work-related changes (see Table 1 for full items, response options, and item descriptives) [24]. Physical distancing was measured with the item, “How much are you practicing physical distancing (including self-quarantining, sheltering-in-place, or staying at home)?” Higher scores reflect increased practices of physical distancing. Engagement in outdoor activities was measured with the item, “How often are you getting outside of your house for allowed shelter-in-place activities (e.g., going on a walk or a run, walking a pet, spending time in your backyard)?” Higher scores reflect increased practices of engagement in safe outdoor shelter-in-place activities. Work-related changes were measured with the dichotomous item, “Have you experienced Coronavirus-related work changes?” 

The Difficulties in Emotion Regulation Scale (DERS) for COVID-19 Pandemic [24] was modified from the DERS Short Form (DERS-SF) [22] to capture difficulties in emotion regulation during the COVID-19 pandemic. This scale consists of eighteen items (e.g., “I have no idea how I am feeling”). The original scale opening prompt of “when I am upset” was replaced with “during the pandemic” to reflect boredom experiences in the COVID-19 pandemic. Higher scores reflect lower emotion regulation. Reliability of the DERS-SF is good and consistent with prior reports (0.69–0.80) [22].

A hierarchical regression predicting the role of boredom proneness on COVID-19 pandemic emotion regulation difficulties was conducted. In step 1, boredom proneness was regressed onto control variables including age and sex. In step 2, boredom proneness was regressed onto boredom COVID-19 related lifestyle changes of physical distancing, engagement in safe outdoor shelter-in-place activities, and work-related changes. In step 3, boredom proneness was regressed onto COVID-19 pandemic emotion regulation difficulties (the target predictor variables). The change in R2 associated with each step was evaluated to determine whether the entry of the predictor(s) significantly improved the model. Partial correlation (pr) was computed to determine the strength of the relationship between each independent variable and dependent variable after adjusting for other variables in the model. The multiple coefficient of determination (R2) was used to describe the percent variation accounted for in the dependent variable by the independent variables in the model.

## 3. Results

Correlations among all study variables are presented in Table 2. Boredom proneness was strongly positively correlated to COVID-19 pandemic emotion regulation difficulties.

In our final model, age, sex, COVID-19 lifestyle change items, and COVID-19 pandemic emotion regulation difficulties account for 35.9% of the variability in boredom proneness. As shown in Table 3, results revealed the addition of COVID-19 pandemic emotion regulation difficulties to the model significantly improved the prediction accuracy of the model, ∆R^2 = 0.28, ∆F(1,51) = 22.43, *p* < 0.001. Therefore, COVID-19 pandemic emotion regulation difficulties predict boredom proneness, over and above, the potentially confounding influence of age, sex, and COVID-19 lifestyle change items. More specifically, the final model revealed that COVID-19 pandemic emotion regulation difficulties, b = 0.94, $\;SE_{b\;}$ = 0.20, β = 0.54, t(51) = 4.74, *p* < 0.001, was the only significant predictor of boredom proneness. Specifically, for every one unit increase in COVID-19 pandemic emotion regulation difficulties, an increase of 0.94 units in boredom proneness is predicted. The partial correlation between COVID-19 pandemic emotion regulation difficulties and boredom proneness, with the influence of age, sex, and COVID-19 lifestyle change items controlled, is 0.55.

## 4. Discussion

The onset of the COVID-19 pandemic had an unprecedented impact on people’s environment and daily life. One day people were at school or work with regular social interactions and access to recreation and entertainment. The next day people were at home isolated from others with ordinary daily routines restricted for an unknown period. Early in the pandemic, individuals responded to restrictions with reports of feeling, or expecting to feel, bored. A decrease in the familiar environmental stimulation and loss of autonomy to create stimulation may alone be sufficient to induce boredom more frequently. We hypothesized individual differences in the ability to effectively regulate negative emotions would play an important role in resolving boredom early in the pandemic. We tested this hypothesis by measuring emotion regulation difficulties and boredom proneness specifically as experienced during the COVID-19 pandemic. We expected more emotion regulation difficulties to relate to more boredom proneness. Consistent with this expectation, the main finding was higher levels of emotion regulation difficulties predicted higher levels of boredom proneness above and beyond demographic variables and items designed to measure COVID-19 lifestyle changes such as adherence to restrictions and engaging in activity outside of the home. 

These findings add to the growing body of literature on COVID-19 and boredom. Studies from across the world found increases in boredom proneness [25,26] some within specific sub-groups (e.g., those who reduced physical activity) [27] and implications for boredom in COVID-19 related factors such as compliance with restrictions [28] or coping style [29]. However, these studies predominantly focused on adults. An important contribution of the current study is it examined boredom proneness in college students who shared a similar pre-pandemic environment while living and attending class on campus. Their pre-pandemic environments were structured to facilitate social interactions, provide access to recreation, and required traveling to and from locations such as home, school, work, entertainment, etc. At the onset of the pandemic, on 23 March 2020, these students transitioned to a remote learning environment where local and state restrictions on face-to-face interactions and access to many forms of recreation (e.g., movies, shopping) were in place. It was during this early period of the pandemic the data reported here were collected. The widespread experience of boredom at the onset of the pandemic may be due to these autonomy controlling restrictions and similar alterations to people’s everyday environments. This possibility is consistent with the MAC model proposed by Westgate and Wilson [10] which posits boredom can arise when people are under-stimulated and demands on attention are inadequate as well as prior research on how reductions in autonomy are associated with greater boredom [20]. 

Internal factors, like emotion regulation, also contribute to boredom. In the BFM [12], for example, boredom is construed as an emotion that serves as a cue to redirect attention [30]. If a more satisfying activity is identified, boredom is mitigated. Prior studies have shown emotion regulatory styles, such as sensitivity to punishment, relate to boredom proneness [8] and lower levels of boredom proneness relate to a more adaptive physiological response during a lab task designed to induce boredom [17]. An important contribution of the current study is it shows people who felt they were experiencing more emotion regulation difficulties early in the pandemic also report higher levels of boredom proneness. This adds to the growing body of evidence linking individual differences in emotion regulation to trait boredom but in the specific context of the COVID-19 pandemic.

Boredom proneness is considered to reflect trait boredom, or a stable personality dimension. However, we know personality is not as fixed as previously thought and is influenced by situational factors [31]. Boredom researchers may benefit from more purposefully accounting for the influence of situation on what are historically considered trait measures of boredom. What is more likely are individual patterns of response across situations [31]. For example, one may experience greater synchronicity with difficulties in emotion regulation and experiences of boredom when alone versus when socializing. These types of questions about patterns of behavior require collection of longitudinal data to capture contributions of individual and situational factors alongside intensity of boring experiences which can inform health promotion efforts.

Despite the known associations between boredom and negative outcomes, few interventions explicitly target boredom [32]. Educational classroom settings, such as those found at universities, serve to reduce student autonomy. Given the association between autonomy constraining environments and boredom, this topic is especially important to address on college campuses. A recent study found a psychoeducational brief video was effective in teaching college students about boredom [33]. One practical implication of the current study is the understanding that some types of students are more prone to experiencing boredom. Interventions, like the psychoeducational video, could screen for things like difficulties with emotion regulation or dislike of the experience of boredom [34], and provide a tailored intervention. Currently, efforts supporting young adults to effectively cope with boredom are lacking. It is time for the growth in boredom theory and basic and applied research to translate into developing, implementing, and evaluating boredom coping interventions.

A number of limitations should be considered when interpreting study findings. First, difficulties in emotion regulation were captured specifically in reference to the COVID-19 pandemic. Although taken from a validated survey, these adapted measures have not been fully tested for reliability and validity. The context specific nature of these measures also means it is unclear whether they reflect more stable individual difficulties as compared to situational emotion regulation difficulties. We recommend future studies account for individual differences in trait emotion regulation through longitudinal data collection to determine causal processes at play and, as previously suggested, separation of individual and situational components. Second, the current study leveraged access from a prior study, completed pre-pandemic, to quickly launch the survey early in the pandemic. Unfortunately, enrollment was less than anticipated, even with incentives, resulting in a relatively small sample size compared to the original study. Third, data were from college students at a large public university in the northwest and may not apply to other developmental stages or higher education institutions or settings. 

## 5. Conclusions

Despite these limitations, findings from the current study further our understanding of the impacts of COVID-19 on college students’ experiences of boredom. As greater calls are made to support effective boredom coping generally and in educational settings, [35,36] findings, such as those from the current study, are critical to the development of such coping strategies. The current study suggests students who struggle with emotion regulation are more likely to experience boredom proneness early in the COVID-19 pandemic. Although the COVID-19 pandemic is a unique environmental factor, external or situational influences significantly impact individual experience. If we can develop tools to support individuals in navigating an extreme event like this, they likely will translate to more common, daily environmental constraints. 

## Figures and Tables

**Table 1:** Descriptive statistics for study measures.

Construct	Scale/Items	Response Options	Mean	Range	Standard Deviation	Reliability (α)
Boredom Proneness	Short Boredom Proneness Scale	1 = strongly disagree to 7 = strongly agree	3.57	1.13–6.00	1.21	0.88
COVID-19 Related Lifestyle Changes	How much are you practicing physical distancing (including self-quarantining, sheltering-in-place, or staying at home)?	1 = “None of the time. I am continuing my normal daily schedule.” to 4 = “All of the time. I am staying home almost all of the time.	3.28	2.00–4.00	0.56	n/a
	How often are you getting outside of your house for allowed shelter-in-place activities (e.g., going on a walk or a run, walking a pet, spending time in your backyard)?	1 = less than once a week to 5 = multiple times a day	3.09	1.00–5.00	1.16	n/a
	Have you experienced Coronavirus-related work changes?	0 = no work changes and 1 = work changes	0.50	0.00–1.00	0.50	n/a
Emotion Regulation	Difficulties in Emotion Regulation Scale for COVID-19 Pandemic	1 = almost never to 5 = almost always	2.49	1.06–3.89	0.69	0.89

**Table 2:** Correlations Matrix for all study variables.

	2.	3.	4.	5.	6.	7.
1. Age	0.01	−0.11	0.00	0.01	−0.08	−0.08
2. Sex		−0.01	−0.11	0.16	0.12	−0.06
3. COVID-19 lifestyle change: physical distancing			−0.04	<0.00	0.03	−0.13
4. COVID-19 lifestyle change: Engagement in safe outdoor shelter-in-place activities				−0.11	−0.12	−0.21
5. COVID-19 lifestyle change: Work-related changes					0.01	0.01
6. COVID-19 pandemic emotion regulation difficulties						0.54 **
7. Boredom proneness						

Note. *N* = 58. ** *p* < 0.01.

**Table 3:** Hierarchical regression predicting boredom proneness.

**Step 1**					
	**Model Statistics**				
	*R* ^2^	*F*	*p*		
	0.01	0.27	0.77		
Predictors	Predictor Statistics				
	β	*b*	*SE_b_*	*t*	*p*
Age	−0.08	−0.06	0.11	−0.59	0.56
Sex	−0.06	−0.21	0.49	−0.42	0.68
**Step 2**					
	**Model Statistics**				
	Δ*R*^2^	Δ*F*	*p*		
	0.07	1.28	0.29		
Predictors	Predictor Statistics				
	β	*b*	*SE_b_*	*t*	*p*
Age	−0.10	−0.07	0.10	−0.71	0.48
Sex	−0.08	−0.30	0.50	−0.60	0.55
COVID-19 lifestyle change: physical distancing	−0.14	−0.32	0.29	−1.08	0.28
COVID-19 lifestyle change: Engagement in safe outdoor shelter-in-place activities	−0.23	−0.24	0.14	−1.67	0.10
COVID-19 lifestyle change: Work-related changes	−0.003	−0.01	0.32	−0.02	0.98
**Step 3**					
	**Model Statistics**				
	Δ*R*^2^	Δ*F*	*p*		
	0.28	22.43	<0.001 ***		
Predictors	Predictor Statistics				
	β	*b*	*SE_b_*	*t*	*p*
Age	−0.05	−0.04	0.09	−0.48	0.63
Sex	−0.14	−0.52	0.42	−1.22	0.23
COVID-19 lifestyle change: physical distancing	−0.16	−0.34	0.25	−1.38	0.17
COVID-19 lifestyle change: Engagement in safe outdoor shelter-in-place activities	−0.17	−0.17	0.12	−1.46	0.15
COVID-19 lifestyle change: Work-related changes	0.01	0.02	0.27	0.08	0.94
COVID-19 pandemic emotion regulation difficulties	0.54	0.94	0.20	4.74	<0.001 ***

Note: *** *p* < 0.001.

## Data Availability

Study data are available upon request.

## Supplementary Materials

The following supporting information can be downloaded at: https://www.mdpi.com/article/10.3390/bs12080296/s1, Table S1: Scale items and response options.

## References

[1] (2022). Centers for Disease Control and Prevention CDC Museum COVID-19 Timeline. https://www.cdc.gov/museum/timeline/covid19.html.

[2] Alzueta E., Perrin P., Baker F.C., Caffarra S., Ramos-Usuga D., Yuksel D., Arango-Lasprilla J.C. (2021). How the COVID-19 Pandemic Has Changed Our Lives: A Study of Psychological Correlates across 59 Countries. J. Clin. Psychol..

[3] Ammar A., Mueller P., Trabelsi K., Chtourou H., Boukhris O., Masmoudi L., Bouaziz B., Brach M., Schmicker M., Bentlage E. (2020). Psychological Consequences of COVID-19 Home Confinement: The ECLB-COVID19 Multicenter Study. PLoS ONE.

[4] Knox L., Karantzas G.C., Romano D., Feeney J.A., Simpson J.A. (2022). One Year on: What We Have Learned about the Psychological Effects of COVID-19 Social Restrictions: A Meta-Analysis. Curr. Opin. Psychol..

[5] (2020). Google Trends Explore: Bored. https://trends.google.com/trends/explore?date=2020-01-01%202020-12-31&geo=US&q=bored.

[6] Westgate E.C., Buttrick N., Lin Y., el Helou G., Agostini M., Bélanger J.J., Gützkow B., Kreienkamp J., Abakoumkin G., Hanum Abdul Khaiyom J. Pandemic Boredom: Predicting Boredom and Its Consequences during Self-Isolation and Quarantine. https://psyarxiv.com/78kma.

[7] Eastwood J.D., Frischen A., Fenske M.J., Smilek D. (2012). The Unengaged Mind: Defining Boredom in Terms of Attention. Perspect. Psychol. Sci..

[8] Mercer-Lynn K.B., Bar R.J., Eastwood J.D. (2014). Causes of Boredom: The Person, the Situation, or Both?. Personal. Individ. Differ..

[9] van Tilburg W.A.P., Igou E.R. (2012). On Boredom: Lack of Challenge and Meaning as Distinct Boredom Experiences. Motiv. Emot..

[10] Westgate E.C., Wilson T.D. (2018). Boring Thoughts and Bored Minds: The MAC Model of Boredom and Cognitive Engagement. Psychol. Rev..

[11] Bench S.W., Lench H. (2013). On the Function of Boredom. Behav. Sci..

[12] Tam K.Y.Y., van Tilburg W.A.P., Chan C.S., Igou E.R., Lau H. (2021). Attention Drifting in and out: The Boredom Feedback Model. Personal. Soc. Psychol. Rev..

[13] Chin A., Markey A., Bhargava S., Kassam K.S., Loewenstein G. (2017). Bored in the USA: Experience Sampling and Boredom in Everyday Life. Emotion.

[14] Martz M.E., Schulenberg J.E., Patrick M.E., Kloska D.D. (2018). “I Am so Bored!”: Prevalence Rates and Sociodemographic and Contextual Predictors of Boredom among American Adolescents. Youth Soc..

[15] Miller J.A., Caldwell L.L., Weybright E.H., Smith E.A., Vergnani T., Wegner L. (2014). Was Bob Seger Right? Relation between Boredom in Leisure and [Risky] Sex. Leis. Sci..

[16] Farmer R., Sundberg N.D. (1986). Boredom Proneness: The Development and Correlates of a New Scale. J. Personal. Assess..

[17] Perone S., Weybright E.H., Anderson A.J. (2019). Over and over Again: Changes in Frontal EEG Asymmetry across a Boring Task. Psychophysiology.

[18] Weybright E.H., Caldwell L.L., Ram N., Smith E.A., Wegner L. (2015). Boredom Prone or Nothing to Do? Distinguishing between State and Trait Leisure Boredom and Its Association with Substance Use in South African Adolescents. Leis. Sci..

[19] Ryan R.M., Deci E.L. (2000). Self-Determination Theory and the Facilitation of Intrinsic Motivation, Social Development, and Well-Being. Am. Psychol..

[20] van Hooft E.A.J., van Hooff M.L.M. (2018). The State of Boredom: Frustrating or Depressing?. Motiv. Emot..

[21] Baumeister R.F., Vohs K.D., Leary M.R., Tangney J.P. (2011). Self-Regulation and the Executive Function of the Self. Handbook of Self and Identity.

[22] Kaufman E.A., Xia M., Fosco G., Yaptangco M., Skidmore C.R., Crowell S.E. (2016). The Difficulties in Emotion Regulation Scale Short Form (DERS-SF): Validation and Replication in Adolescent and Adult Samples. J. Psychopathol. Behav. Assess..

[23] Struk A.A., Carriere J.S.A., Cheyne J.A., Danckert J. (2017). A Short Boredom Proneness Scale. Assessment.

[24] Pfeifer J.H., Ladouceur C.D., Byrne M.L., Flannery J.E., Chavez S., Cheng T.W., Flournoy J.C., Oosterhoff B. (2021). Assessment of COVID-19 Experiences (ACE) for Adolescents—Research Tracker and Facilitator. https://osf.io/py7vg/.

[25] Boateng G.O., Doku D.T., Enyan N.I.E., Owusu S.A., Aboh I.K., Kodom R.V., Ekumah B., Quansah R., Boamah S.A., Obiri-Yeboah D. (2021). Prevalence and Changes in Boredom, Anxiety and Well-Being among Ghanaians during the COVID-19 Pandemic: A Population-Based Study. BMC Public Health.

[26] Wessels M., Utegaliyev N., Bernhard C., Welsch R., Oberfeld D., Thönes S., von Castell C. (2022). Adapting to the Pandemic: Longitudinal Effects of Social Restrictions on Time Perception and Boredom during the Covid-19 Pandemic in Germany. Sci. Rep..

[27] McCurdy A., Stearns J.A., Rhodes R.E., Hopkins D., Mummery K., Spence J.C. (2022). Relationships Between Physical Activity, Boredom Proneness, and Subjective Well-Being Among U.K. Adults During the COVID-19 Pandemic. J. Sport Exerc. Psychol..

[28] Boylan J., Seli P., Scholer A.A., Danckert J. (2021). Boredom in the COVID-19 Pandemic: Trait Boredom Proneness, the Desire to Act, and Rule-Breaking. Personal. Individ. Differ..

[29] Waterschoot J., van der Kaap-Deeder J., Morbée S., Soenens B., Vansteenkiste M. (2021). “How to Unlock Myself from Boredom?” The Role of Mindfulness and a Dual Awareness- and Action-Oriented Pathway during the COVID-19 Lockdown. Personal. Individ. Differ..

[30] Wojtowicz Z., Loewenstein G. (2020). Curiosity and the Economics of Attention. Curr. Opin. Behav. Sci..

[31] Ram N., Morelli S., Lindberg C., Carstensen L.L., Schaie K., Abeles R.P. (2008). From Static to Dynamic: The Ongoing Dialectic about Human Development. Social Structures and Aging Individuals.

[32] Caldwell L.L., Smith E.A., Wegner L., Vergnani T., Mpofu E., Flisher A.J., Mathews C. (2004). Health Wise South Africa: Development of a Life Skills Curriculum for Young Adults. World Leis. J..

[33] Parker P.C., Tze V.M.C., Daniels L.M., Sukovieff A. (2021). Boredom Intervention Training Phase I: Increasing Boredom Knowledge through a Psychoeducational Video. Int. J. Environ. Res. Public Health.

[34] Tam K.Y.Y., Chan C.S., van Tilburg W.A.P., Lavi I., Lau J.Y.F. (2022). Boredom Belief Moderates the Mental Health Impact of Boredom among Young People: Correlational and Multi-wave Longitudinal Evidence Gathered during the COVID-19 Pandemic. J. Personal..

[35] Daniels L.M., Tze V.M.C., Goetz T. (2015). Examining Boredom: Different Causes for Different Coping Profiles. Learn. Individ. Differ..

[36] Nett U.E., Goetz T., Hall N.C. (2011). Coping with Boredom in School: An Experience Sampling Perspective. Contemp. Educ. Psychol..

