# Supplementary material for: Difficulties with Emotion Regulation during COVID-19 and Associations with Boredom in College Students
Source: Behav Sci (Basel). 2022 Aug 20;12(8):296. doi: 10.3390/bs12080296 (PMC9404998; doi:10.3390/bs12080296)
Supplement: Supplementary file 1 [file behavsci-12-00296-s001.zip › behavsci-1841682-supplementary.pdf]

**Table S1.** Scale items and response options.

| | |
|---|---|
| **Short Boredom Proneness Scale** [23] | Stem: Thinking of yourself over the past two weeks, select the phrase that best describes the degree to which you agree or disagree with each statement.<br>1. I often find myself at "loose ends," not knowing what to do.<br>2. I find it hard to entertain myself.<br>3. Many things I have to do are repetitive and monotonous.<br>4. It takes more stimulation to get me going than most people.<br>5. I don't feel motivated by most things that I do.<br>6. In most situations, it is hard for me to find something to do or see to keep me interested.<br>7. Much of the time, I just sit around doing nothing.<br>8. Unless I am doing something exciting, even dangerous, I feel half-dead and dull.<br><br>Response options: 1=Strongly disagree; 2=Disagree; 3=Somewhat disagree; 4=Neither agree nor disagree; 5=Somewhat agree; 6=Agree; 7=Strongly agree |
| **COVID-19 Related Lifestyle Changes** [24] | How much are you practicing physical distancing (including self-quarantining, sheltering-in-place, or staying at home)?<br><br>Response options: 1=None of the time. I am continuing my normal daily schedule.; 2=Some of the time. I have reduced some of the time that I am in public spaces or gatherings.; 3=Most of the time. I only leave for food, doctor appointments, and other essentials.; 4=All of the time. I am staying home almost all of the time. |
| | How often are you getting outside of your house for allowed shelter-in-place activities (e.g., going on a walk or a run, walking a pet, spending time in your backyard)?<br><br>Response options: 1=Less than once a week; 2=Once a week; 3=Every couple of days; 4=Once a day; 5=Multiple times a day |
| | Have you experienced Coronavirus-related work changes?<br><br>Response options coded as: 0=no work related changes; 1=work changes |
| **Difficulties in Emotion Regulation Scale - Short Form (DERS-SF)** [22,24] | *Scale was reworded to "During the pandemic" vs. "When I'm upset"*<br>Please indicate how often the following apply to you.<br>1. During this pandemic, I am paying attention to how I feel.*<br>2. During this pandemic, I have no idea how I am feeling.<br>3. During this pandemic, I am having difficulty making sense out of my feelings.<br>4. During this pandemic, I care about what I am feeling.*<br>5. During this pandemic, I am confused about how I feel.<br>6. During this pandemic, I am acknowledging my emotions.*<br>7. During this pandemic, I am embarrassed for feeling the way I do.<br>8. During this pandemic, I am having difficulty getting work done.<br>9. During this pandemic, I have become out of control.<br>10. During this pandemic, I believe that I will end up feeling very depressed.<br>11. During this pandemic, I am having difficulty focusing on other things.<br>12. During this pandemic, I feel guilty for feeling the way I do.<br>13. During this pandemic, I am having difficulty concentrating.<br>14. During this pandemic, I am having difficulty controlling my behaviors.<br>15. During this pandemic, I believe that there is nothing I can do to make myself feel better. |

| | |
|---|---|
| | 16. During this pandemic, I am becoming irritated at myself for feeling the way I do.<br>17. During this pandemic, I am losing control over my behavior.<br>18. During this pandemic, it is taking a long time to feel better.<br><br>Response options: 1=Almost never; 2=Sometimes; 3=Half the time; 4=Most of the time; 5=Almost aways<br>*=reverse coded |
